# Supplementary material for: Conditional chemoconnectomics (cCCTomics) as a strategy for efficient and conditional targeting of chemical transmission
Source: eLife. 2024 Apr 30;12:RP91927. doi: 10.7554/eLife.91927 (PMC11060718; doi:10.7554/eLife.91927)
Supplement: Figure 3—source data 3. [file elife-91927-fig3-data3.zip › Figure 3 source data3 part 1.zip!/Figure 3-Source data 3-part1/1071f2-1-CRISPResso_Report_ztwktvfp.zip!/CRISPResso_on_1071f2_2/3a.Indel_size_distribution.pdf]

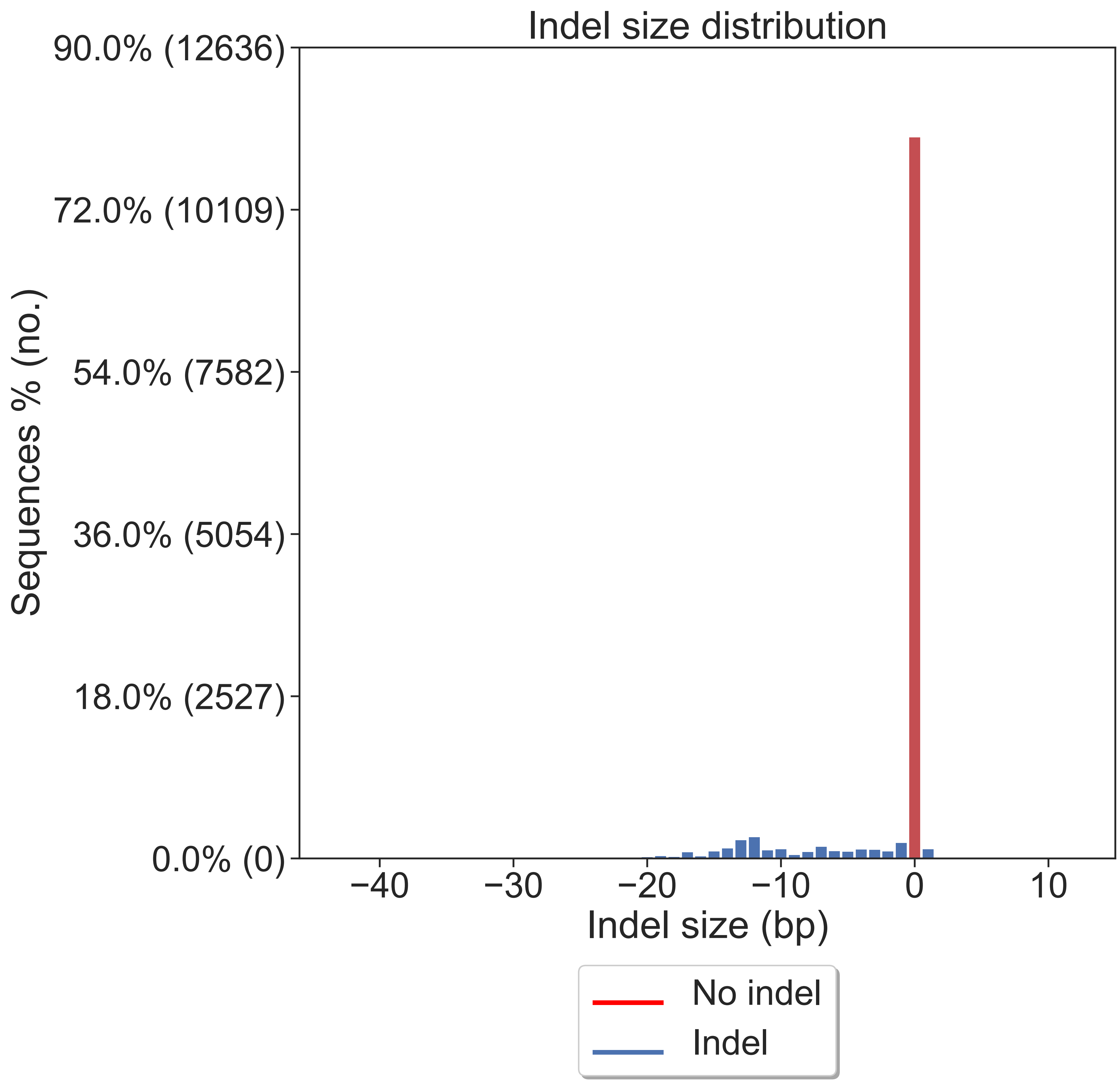
Indel size distribution
90.0% (12636)
72.0% (10109)
54.0% (7582)
36.0% (5054)
18.0% (2527)
0.0% (0)
Sequences % (no.)
−40
−30
−20
−10
0
10
Indel size (bp)
No indel
Indel
